# Supplementary figures and images for: A pan-orthohantavirus human lung xenograft mouse model and its utility for preclinical studies
Source: PLoS Pathog. 2025 Jan 22;21(1):e1012875. doi: 10.1371/journal.ppat.1012875 (PMC11774489; doi:10.1371/journal.ppat.1012875)

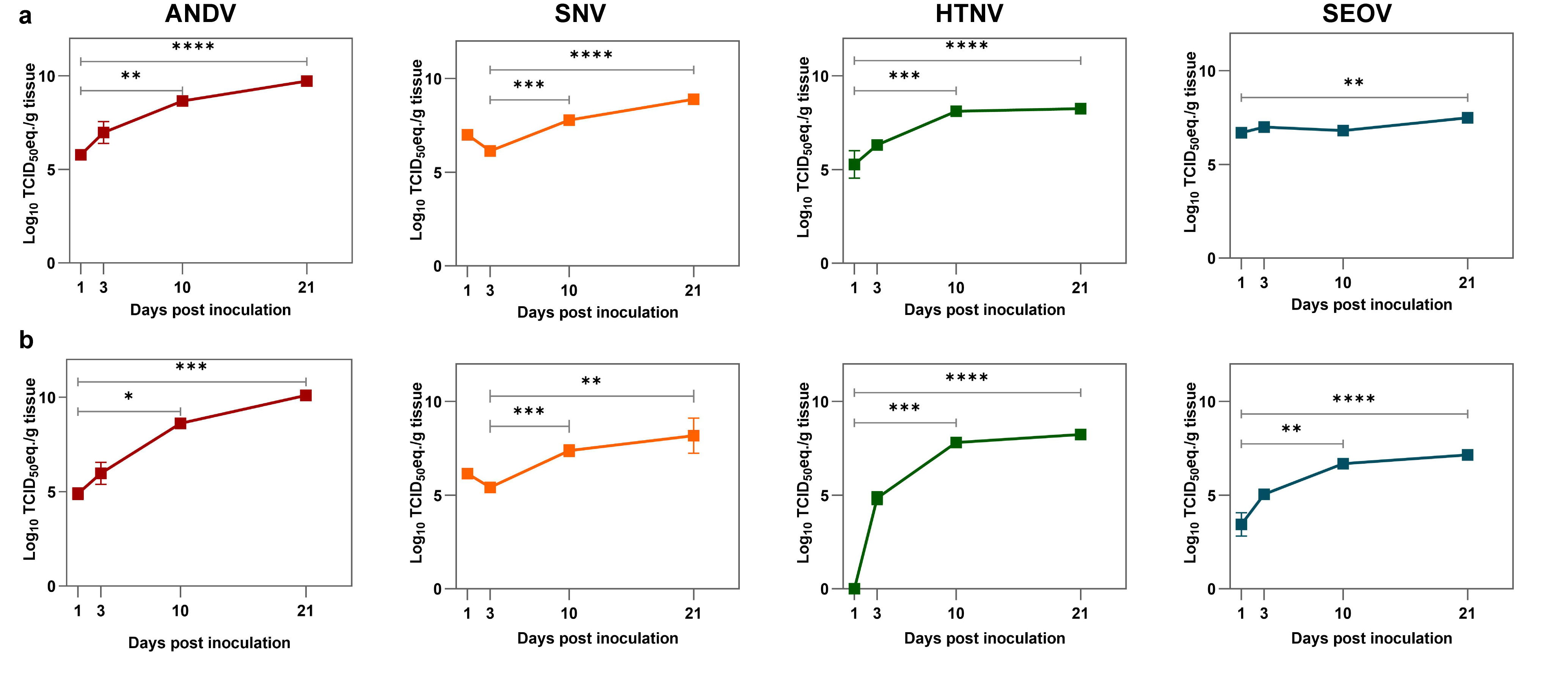

Supplement: S1 Fig — a) Orthohantavirus small (S) segment RNA loads were quantified in human lung xenografts that were directly inoculated with ANDV, SNV, HTNV and SEOV by RT-qPCR. b) Orthohantavirus S segment RNA loads in non-inoculated xenografts were quantified by RT-qPCR. Squares indicate the mean TCID50 equivalent per gram tissue and error bars represent the standard error of the mean. RNA loads in directly inoculated xenografts (a) and non-inoculated xenografts (b) were compared to the lowest RNA load during the course of infection, i.e., 1 or 3 days post inoculation (dpi) by Kruskall-Wallis test with Dunn’s multiple comparisons test. *p < 0.05, **p < 0.005, ***p < 0.001, ****p < 0.0001. Six animals were included per virus per time point. (TIF) [file ppat.1012875.s001.tif]

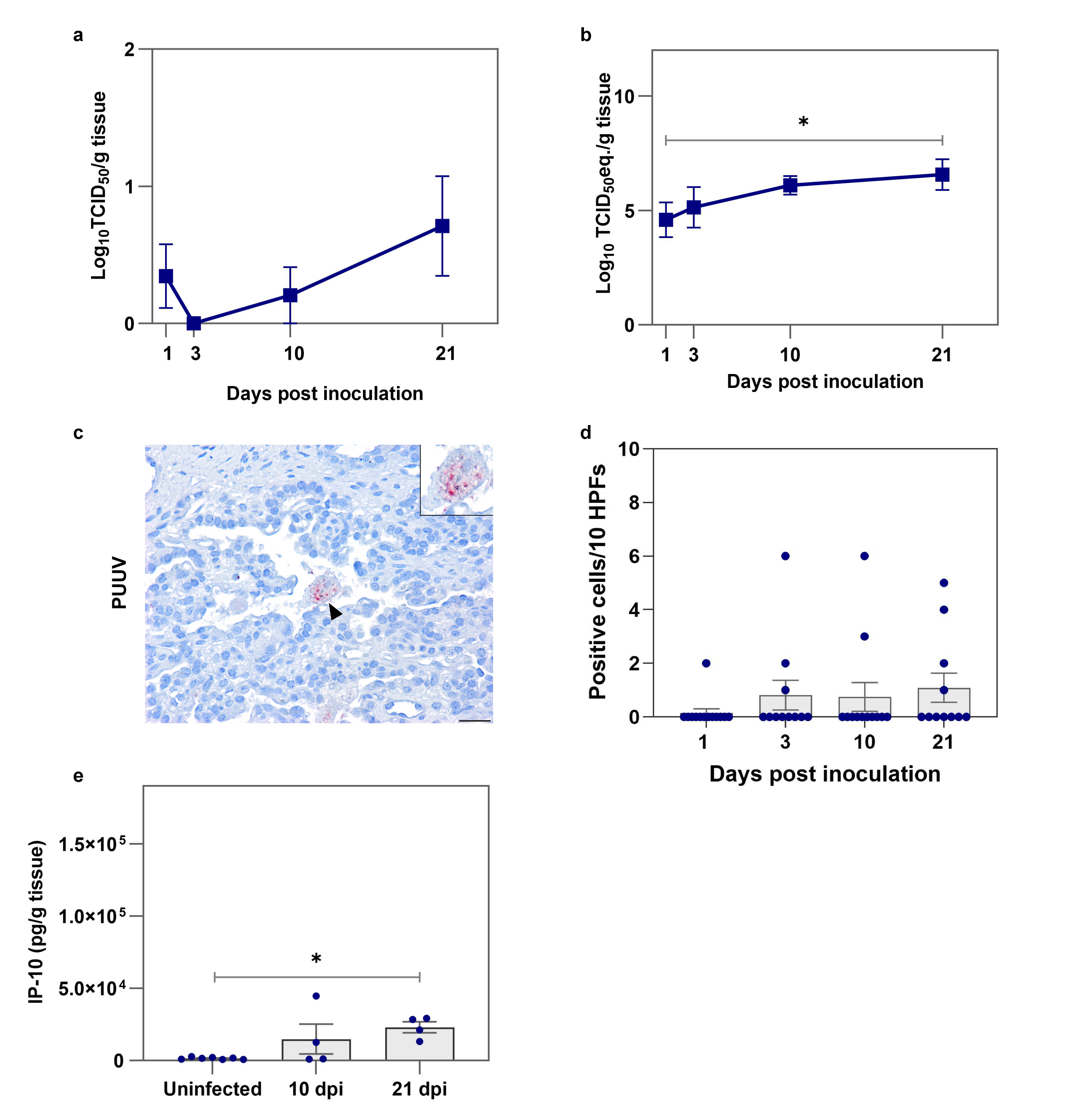

Supplement: S2 Fig — a) Infectious viral titers were quantified in human lung xenografts. Squares indicate the mean TCID50 per gram tissue and error bars represent the standard error of the mean. b) PUUV S segment RNA loads were quantified in human lung xenografts with RT-qPCR. No viremia was detected. Squares indicate the mean TCID50 equivalent per gram tissue and error bars represent the standard error of the mean. c) PUUV nucleoprotein (N) was detected via immunohistochemistry. A representative image is shown for infection at 21 days post inoculation (dpi). Presence of virus antigen is indicated by an arrow head. Scale bar represents 10 µm. Top right inset image offers a zoom-in of an individually infected cell as indicated by the arrow head. d) Quantification of virus antigen detection was performed by counting the number of positive cells for antigen staining per ten high power fields (HPFs). Each circle indicates one evaluated xenograft. Bars represent the mean and error bars represent the standard error of the mean. Infectious viral titers (a), viral RNA loads (b) and number of virus antigen-positive cells (d) in human lung xenografts were compared on 3, 10 and 21 dpi to the infectious viral titers (a), RNA loads (b) or number of virus antigen-positive cells (d) on 1 dpi by Kruskall-Wallis test with Dunn’s multiple comparisons test. e) Human IP-10 levels were measured in tissue homogenates of PUUV-inoculated human lung xenografts. Each circle represents one evaluated xenograft, bars represent the mean and error bars represent the standard error of the mean. IP-10 levels were expressed as picogram cytokine per gram tissue and infected samples were compared to samples from mice that were left uninfected by Kruskall-Wallis test with Dunn’s multiple comparisons test. *p < 0.05. (TIF) [file ppat.1012875.s002.tif]

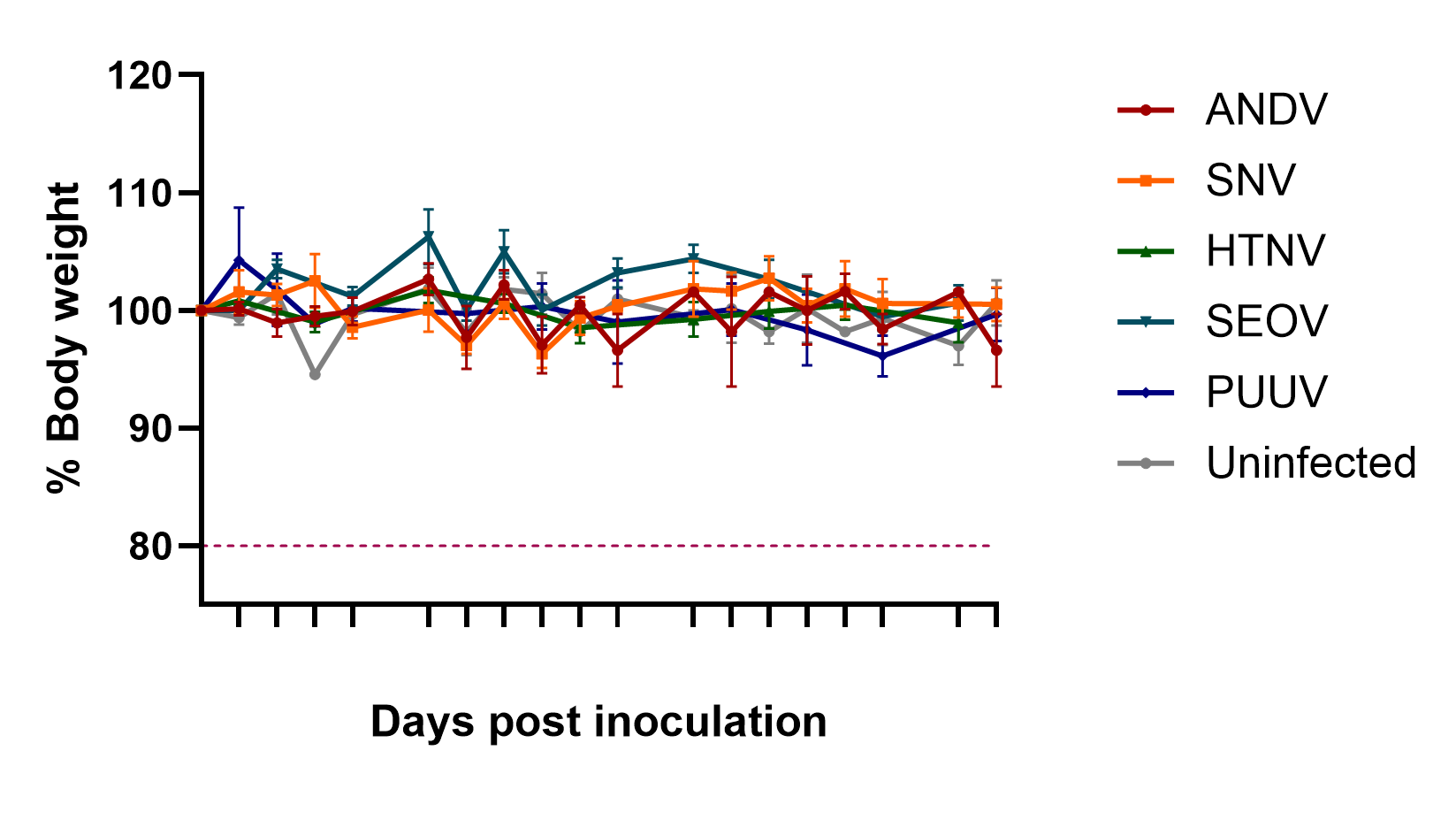

Supplement: S3 Fig — Relative body weight of human lung xenografted mice, following ANDV-, SNV-, HTNV-, SEOV- and PUUV-inoculation. Xenografted mice that were left uninfected were included as an additional control group. The mean of each group is shown as a solid line, error bars represent standard of the mean. The dashed line is indicating the humane endpoint for the total loss of body weight. (TIF) [file ppat.1012875.s003.tif]

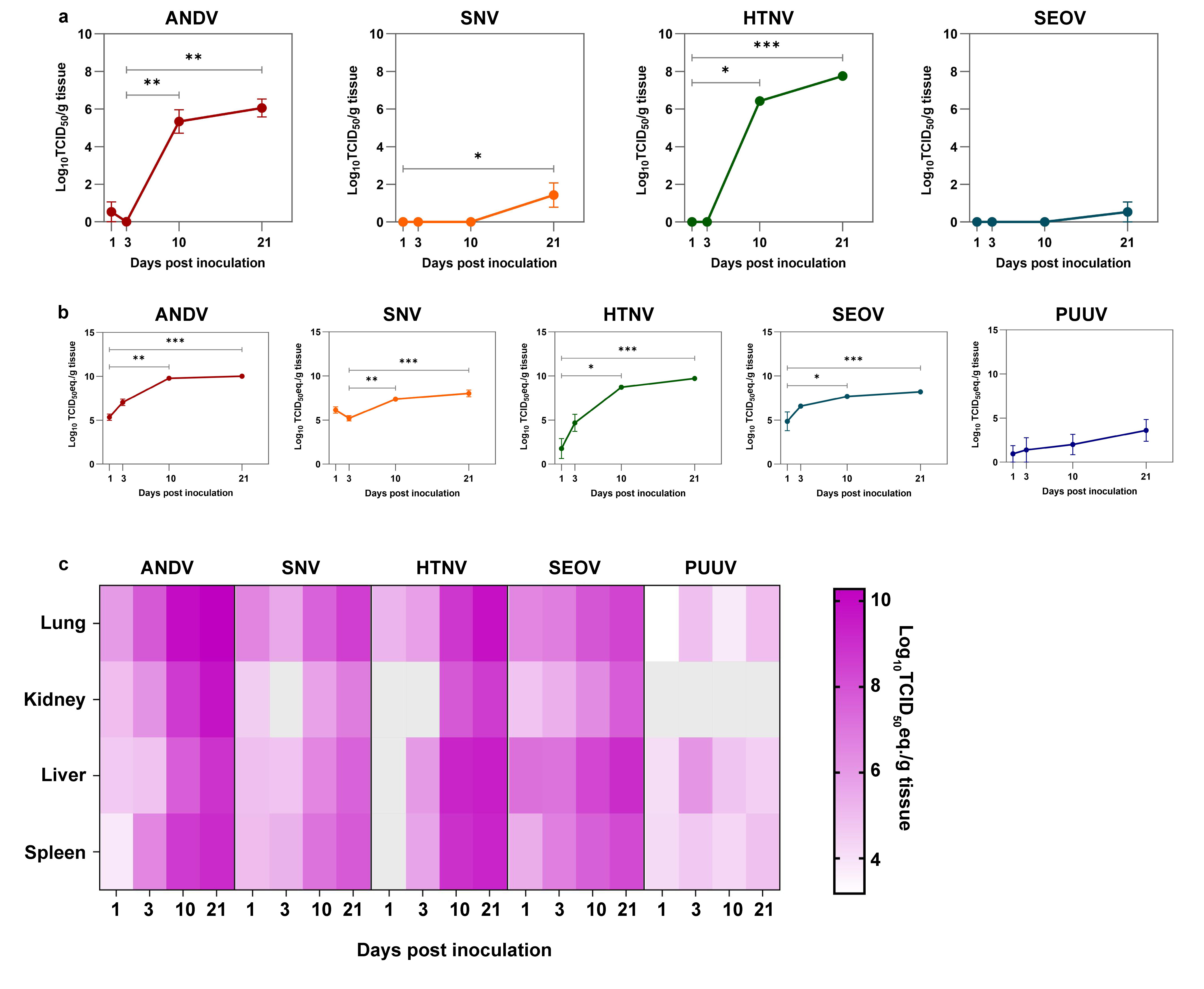

Supplement: S4 Fig — a) Infectious viral titers were quantified in murine lungs of ANDV-, SNV-, HTNV- and SEOV-inoculated animals. No infectious virus was detected in murine lungs of PUUV-inoculated animals. Circles indicate the mean TCID50 per gram tissue and error bars represent the standard error of the mean. b) Orthohantavirus S segment RNA loads were quantified in murine lungs with RT-qPCR. Circles indicate the mean TCID50 equivalent per gram tissue and error bars represent the standard error of the mean. Infectious viral titers (a) and viral RNA loads (b) in murine lungs were compared to the lowest infectious viral titers (a) or RNA loads (b) in murine lungs during the course of infection, i.e., at 1 or 3 dpi by Kruskall-Wallis test with Dunn’s multiple comparisons test. *p < 0.05, **p < 0.005, ***p < 0.001. c) Heatmap displaying quantification of orthohantavirus S segment RNA loads in murine lungs, kidney, liver and spleen as determined by RT-qPCR. Each cell represents the mean per group (N = 6, N = 4 for PUUV) expressed in TCID50 equivalents per gram tissue. Grey cells represent that all samples within a group were below lower limit of detection. (TIF) [file ppat.1012875.s004.tif]

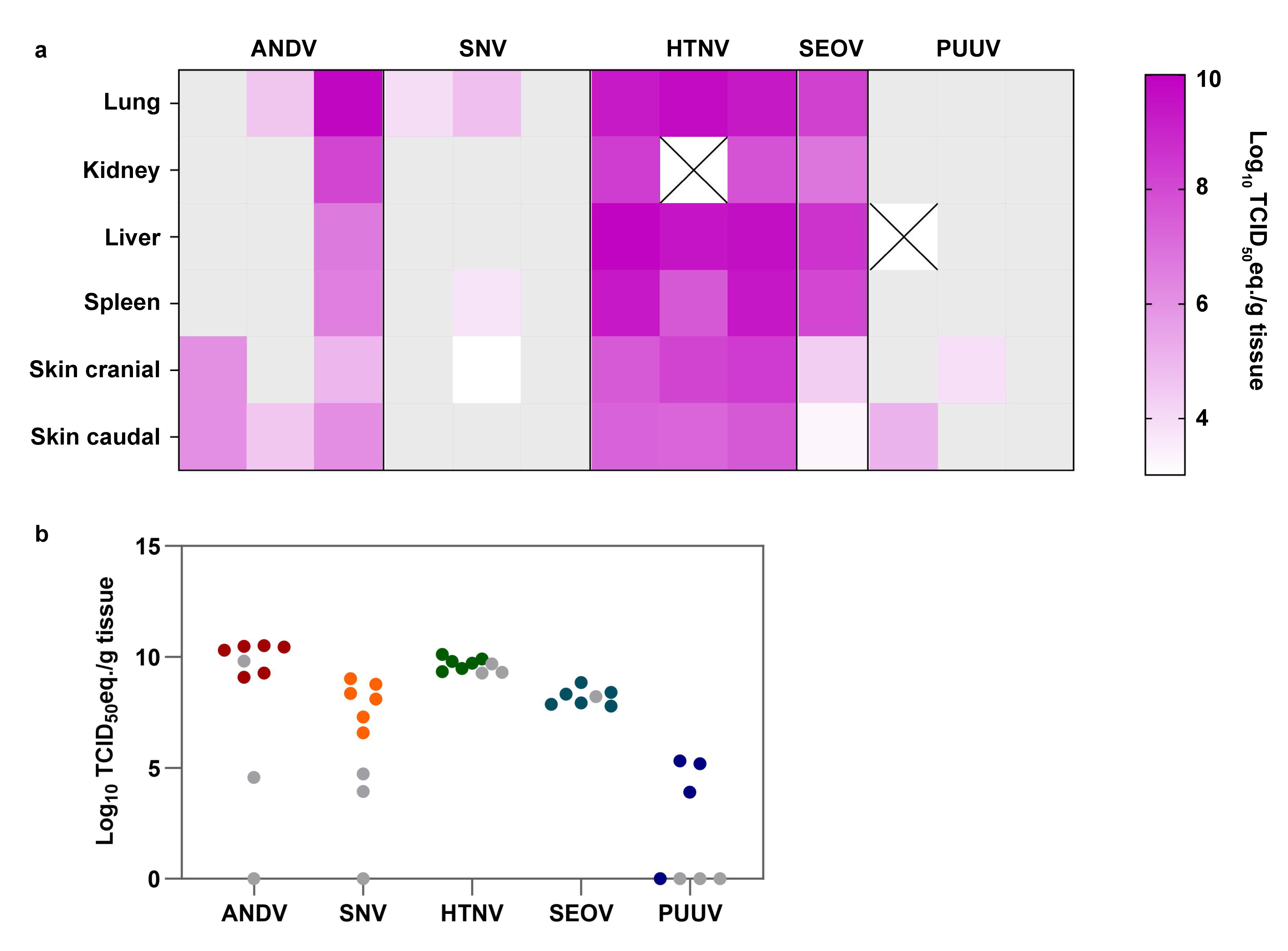

Supplement: S5 Fig — a) Heatmap displaying quantification of orthohantavirus S segment RNA loads in murine lungs, kidney, liver, spleen, cranial and caudal skin as determined by RT-qPCR. Each cell represents the value of each individual animal expressed in TCID50 equivalents per gram tissue. Grey cells represent values below lower limit of detection. X represents undetermined values. b) Comparison of orthohantavirus S segment RNA loads in murine lungs of xenografted and non-grafted NSG mice. Colored circles indicate the values for murine lungs of human lung xenografted animals, whereas grey circles indicate those of non-grafted animals. (TIF) [file ppat.1012875.s005.tif]

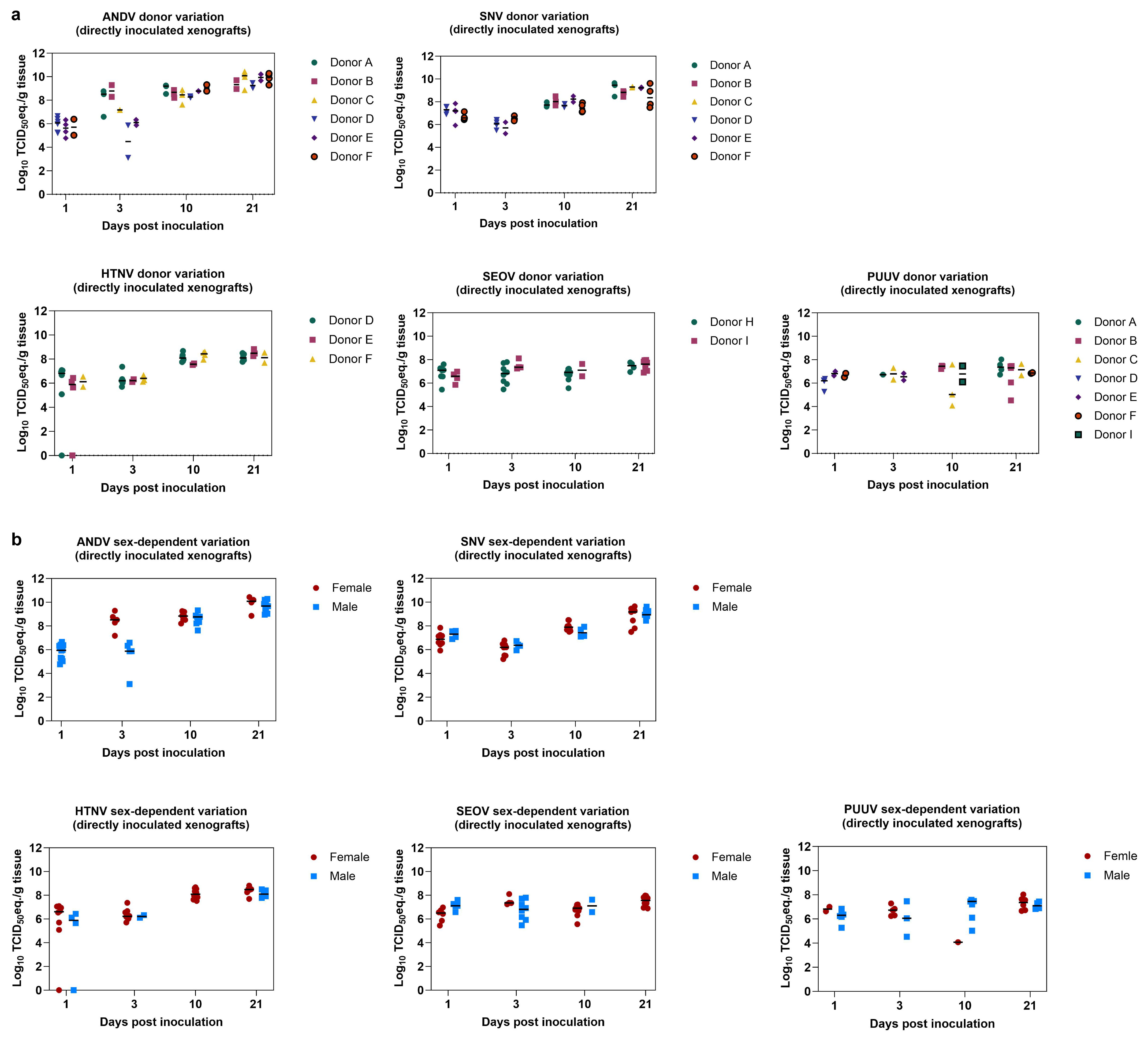

Supplement: S6 Fig — a) Comparison of orthohantavirus S segment RNA loads in directly inoculated human lung xenografts. Results are depicted by human donors and inoculated viruses over time on 1, 3, 10 and 21 days post inoculation and expressed in TCID50 equivalents per gram tissue. Every symbol represents a single human lung xenograft. b) Comparison of orthohantavirus S segment RNA loads in directly inoculated human lung xenografts depicted by the sex of mice and inoculated viruses over time on 1, 3, 10 and 21 days post inoculation. Every symbol represents a single human lung xenograft. (TIF) [file ppat.1012875.s006.tif]

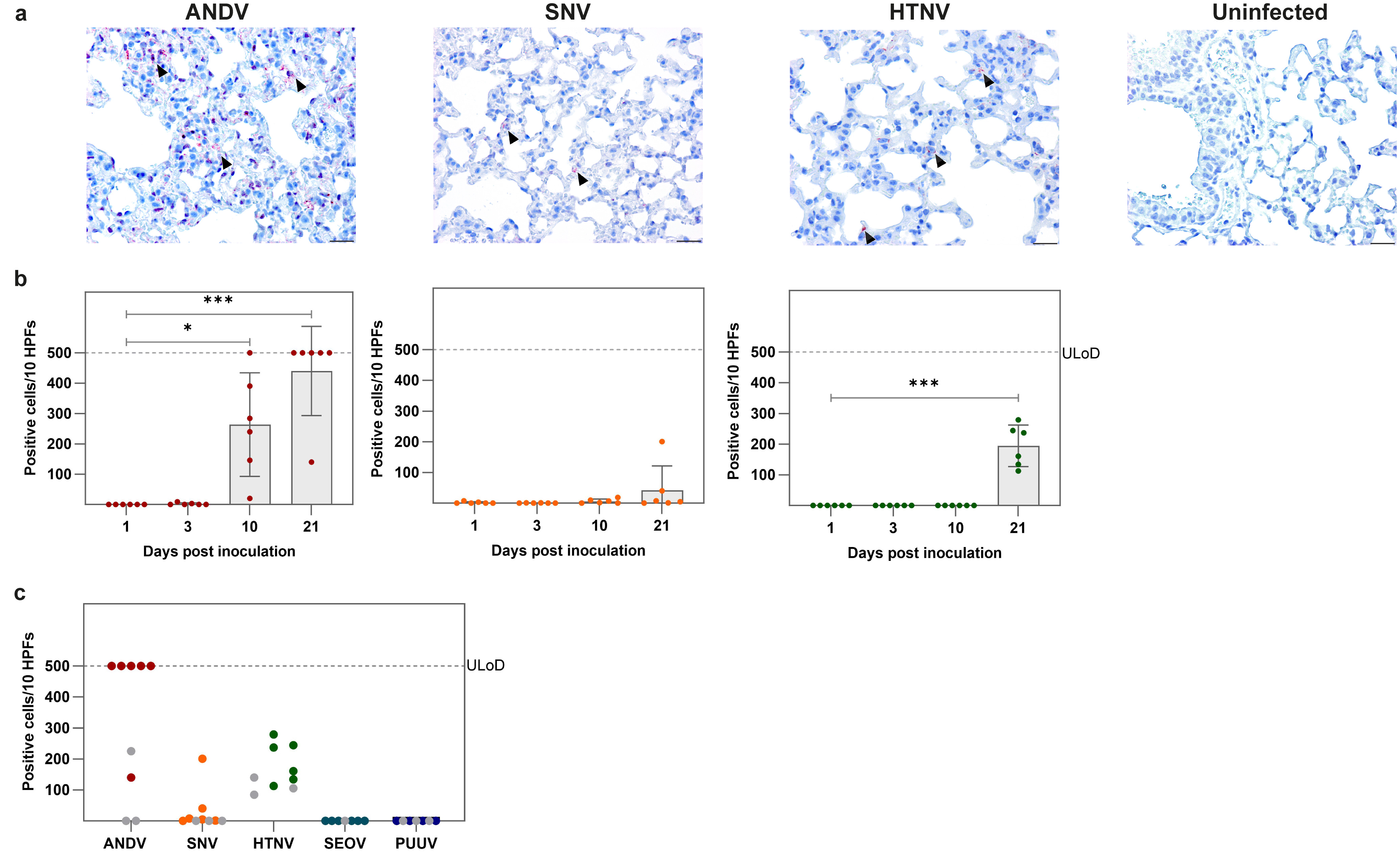

Supplement: S7 Fig — a) Orthohantavirus nucleoprotein (N) was detected via immunohistochemistry. Representative images are shown from the murine lungs of ANDV-, SNV-, and HTNV-inoculated mice at 21 days post inoculation (dpi), together with a representative image of murine lungs from mice that were left uninfected. Scale bars represent 10 µm. Presence of virus antigen is indicated by arrow heads. No virus antigen was detected in the murine lungs of SEOV- and PUUV-inoculated mice. b) Quantification of virus antigen-positive cells was performed by counting the number of positive cells for antigen staining per ten high power fields (HPFs). Each circle represents the murine lungs of one evaluated animal. Bars represent the mean and error bars represent the standard error of the mean. Number of virus antigen-positive cells in murine lungs were compared on 3, 10 and 21 dpi to the number of virus antigen-positive cells on 1 dpi by Kruskall-Wallis test with Dunn’s multiple comparisons test. *p < 0.05, ***p < 0.001. c) Comparison of the number of virus antigen-positive cells in murine lungs of xenografted and non-grafted NSG mice. Colored circles indicate the values for murine lungs of human lung xenografted animals, whereas grey circles indicate those of non-grafted animals. The dashed line indicates upper limit of detection (ULoD). (TIF) [file ppat.1012875.s007.tif]

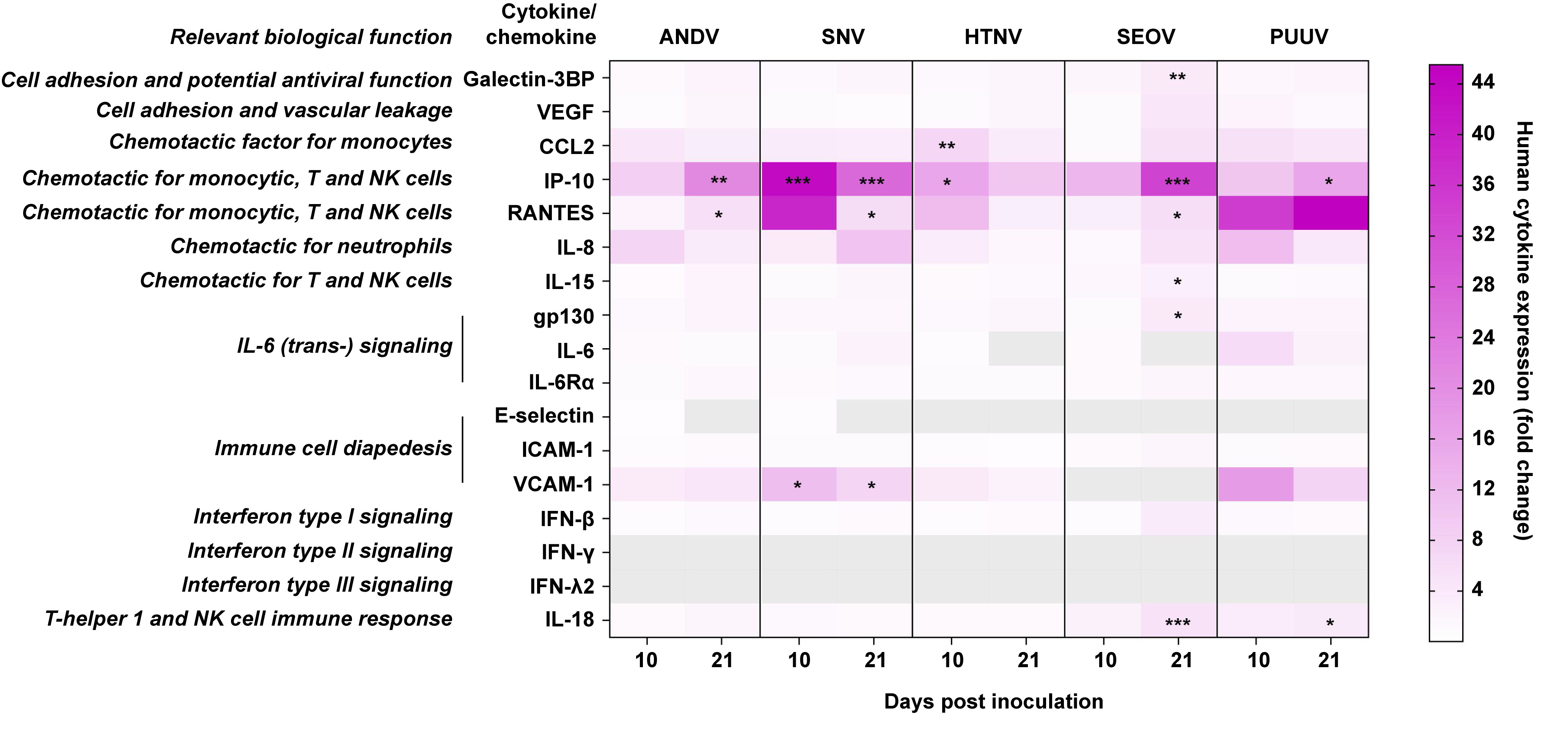

Supplement: S8 Fig — Heatmap displaying quantification of human cytokines and chemokines in human lung xenografts. The potentially relevant biological function of each cytokine and chemokine in context of orthohantavirus infection is indicated on the left. Tissue homogenates weighing less than 10 mg are excluded for analyses, while for inoculated animals only tissue homogenates positive for viral RNA were included. Cytokine levels were expressed as picogram cytokine/chemokine per gram tissue and inoculated samples harvested on 10 and 21 days post inoculation were compared to samples from mice that were left uninfected by Kruskall-Wallis test with Dunn’s multiple comparisons test. Each cell represents the mean fold change of the cytokine/chemokine levels of tissues from inoculated animals over the respective mean levels from uninfected animals. Grey cells represent that all samples within a group were below lower limit of detection. *p < 0.05, **p < 0.005, ***p < 0.001. (TIF) [file ppat.1012875.s008.tif]
