# Supplementary material for: A pan-orthohantavirus human lung xenograft mouse model and its utility for preclinical studies
Source: PLoS Pathog. 2025 Jan 22;21(1):e1012875. doi: 10.1371/journal.ppat.1012875 (PMC11774489; doi:10.1371/journal.ppat.1012875)
Supplement: S1 Table — (DOCX) [file ppat.1012875.s009.docx]

| **Inoculation** |  |  |  |  |  |
| --- | --- | --- | --- | --- | --- |
| **Virus** | **Strain** | **Passage** | **Backtiter mean (TCID_50_/ml)** | **Range** | **Reference** |
| Andes | 9717869 | P+2 | 2.84 x 10^6^ | 1.78 x 10^6^ - 4.64 x 10^6^ | EVAg 002v-EVA400 |
| Sin Nombre | Convict Creek 107 | P+2 | 6.66 x 10^6^ | 3.16 x 10^6^ - 1.00 x 10^7^ | EVAg 002v-EVA401 |
| Hantaan | 76-118 | P+3 | 1.74 x 10^6^ | 3.16 x 10^5^ - 3.16 x 10^6^ | EVAg 008v-EVA1471 |
| Seoul | 80-39 | P+2 | 1.00 x 10^6^ | 1.00 x 10^6^ - 1.00 x 10^6^ | EVAg 008v-EVA1473 |
| Puumala | Cg 18-20 | P+3 | 3.60 x 10^6^ | 5.62 x 10^5^ - 1.00 x 10^7^ | EVAg 007v-00809 |
