# Supplementary material for: A pan-orthohantavirus human lung xenograft mouse model and its utility for preclinical studies
Source: PLoS Pathog. 2025 Jan 22;21(1):e1012875. doi: 10.1371/journal.ppat.1012875 (PMC11774489; doi:10.1371/journal.ppat.1012875)
Supplement: S2 Table — (DOCX) [file ppat.1012875.s010.docx]

| **Virus** | **Xenografted** | **Animals per group (N=)** | **Euthanized at (dpi)** |
| --- | --- | --- | --- |
| Andes | yes | 6 | 1, 3, 10, 21 |
| Sin Nombre | yes | 6 | 1, 3, 10, 21 |
| Hantaan | yes | 6 | 1, 3, 10, 21 |
| Seoul | yes | 6 | 1, 3, 10, 21 |
| Puumala | yes | 4 | 1, 3, 10, 21 |
| Uninfected | yes | 9 | 21 |
| Andes | no | 3 | 21 |
| Sin Nombre | no | 3 | 21 |
| Hantaan | no | 3 | 21 |
| Seoul | no | 1 | 21 |
| Puumala | no | 3 | 21 |
