# Supplementary material for: A pan-orthohantavirus human lung xenograft mouse model and its utility for preclinical studies
Source: PLoS Pathog. 2025 Jan 22;21(1):e1012875. doi: 10.1371/journal.ppat.1012875 (PMC11774489; doi:10.1371/journal.ppat.1012875)
Supplement: S3 Table — (DOCX) [file ppat.1012875.s011.docx]

| **Virus** | **Treatment** | **Animals per group (N=)** | **Euthanized at (dpi)** |
| --- | --- | --- | --- |
| Andes | mAb KL-AN-5E8 | 5 | 3 |
| Andes | mAb CR9114 | 5 | 3 |
| Andes | mAb KL-AN-5E8 | 4 | 10 |
| Andes | mAb CR9114 | 5 | 10 |
