# Supplementary material for: A pan-orthohantavirus human lung xenograft mouse model and its utility for preclinical studies
Source: PLoS Pathog. 2025 Jan 22;21(1):e1012875. doi: 10.1371/journal.ppat.1012875 (PMC11774489; doi:10.1371/journal.ppat.1012875)
Supplement: S4 Table — (DOCX) [file ppat.1012875.s012.docx]

| **Virus** | **Primer/probe** | **5' to 3' sequence** | **Fluorophore and quencher** | **Length (nt)** |
| --- | --- | --- | --- | --- |
| Andes, Sin Nombre | Forward primer | ACACGAACAACAGCTCGTGACT |  | 22 |
|  | Reverse primer | GGTTCAATCCCTGTTGGATCAA |  | 22 |
|  | Taqman probe | CTRCATTGGAGACCAAACTCGGRGAACT | FAM-BHQ | 28 |
| Hantaan | Forward primer | GCTTCTTCCAGATACAGCAGCAG |  | 23 |
|  | Reverse primer | GCCTTTGACTCCTTTGTCTCCAT |  | 23 |
|  | Taqman probe | CCTGCAACAAACAGGGAYTACTTACGGCA | FAM-BHQ | 29 |
| Seoul | Forward primer | GATGAACTGAAGCGCCAACTT |  | 21 |
|  | Reverse primer | CCCTGTAGGATCCCGGTCTT |  | 20 |
|  | Taqman probe | CCGACAGGATTGCAGCAGGGAAGAA | FAM-BHQ | 25 |
| Puumala | Forward primer | AGGCAACAAACAGTGTCAGCA |  | 21 |
|  | Reverse primer | GCATTTACATCAAGGACATTTCCATA |  | 26 |
|  | Taqman probe | CTGACCCGACTGGGATTGAACCTGATG | FAM-BHQ | 27 |
