# Supplementary material for: A pan-orthohantavirus human lung xenograft mouse model and its utility for preclinical studies
Source: PLoS Pathog. 2025 Jan 22;21(1):e1012875. doi: 10.1371/journal.ppat.1012875 (PMC11774489; doi:10.1371/journal.ppat.1012875)
Supplement: Attachment — Submitted filename: Rebuttal Letter_Xenograft.docx [file ppat.1012875.s014.docx]

Melanie Rissmann
Department of Viroscience
Erasmus Medical Center
‘s Gravendijkwal 230
3015 GE Rotterdam
The Netherlands
m.rissmann@erasmusmc.nl

December 31^st^, 2024

Dear Editor,

First of all, we would like to thank you for considering our manuscript in PLOS Pathogens. Furthermore, we would like to thank the reviewers for their useful and constructive comments. All three reviewers acknowledged the high importance and relevance of this study. We believe that the comments have aided to improve the manuscript for the purpose of publication in PLOS Pathogens. Hereby, we would like to summarize our responses to the reviewers comments.

**Part I - Summary**
**Reviewer #1**: Rissman et al present a novel in vivo-model for studies of hantaviruses and for therapeutics against them. The presented model is interesting and can hopefully be of use for the future establishment of treatments, which is highly needed. Strengths: There is a lack of small animal models for hantaviruses, hampering studies of treatment and basic hantavirus research. This model can provide a first step towards such a model by including human lung tissue. Weaknessess: The model is based on immunosuppressed mice, hence important factors contributing to the defence against hantavirus are lacking as are potential immunopathogenic effects. This likely has only a limited impact on studies on for example the effect of anti-hantavirus monoclonal antibodies and on viral replication in human lung cells, but it can limit the possible use of this model for other purposes. Novelty: The xenograft model has previously been used for other viruses, a novelty is that it is here adopted to several different hantaviruses allowing for studies comparing different hantaviruses. General execution and scholarship. The experiments are well designed. Results/data are presented in a clear way in text and figures.

*Author’s response: We thank the reviewer for acknowledging the importance of the study. In correspondence with the reviewers detailed suggestions below, we added more substantial details on existing literature in both introduction and discussion, and limitations of our model in the discussion.*

**Reviewer #2**: The report by Rissmann et al. describes characterization of a lung xenograft mouse model for orthohantaviruses. They show that the subcutaneously grafted tissue can support viral replication, and that virus can spread to other uninoculated graphs as well as murine tissues. The authors also present cytokine analyses which provide some depth to the investigations and appear to correlate with previous literature both in humans and animal models. Importantly, the kinetics of replication (increasing over time until study end) and absence of histopathological findings differentiate these mice from a disease model; the xenograft mouse model presented here is an infection model with the advantage of incorporating human tissue evaluation in a model system. The inclusion of appropriate controls (non-grafted mice) indicating that grafting does not alter overall susceptibility to infection but does increase titers and results in more efficient viral spread further supports use primary as an infection model. However, infection models can serve an important role, particularly when replication can achieve levels that permit more robust evaluation of countermeasures, which is supported by work presented in the report. Overall, the studies are well performed and provide interesting data to the field. It was nice to see both HCPS- and HFRS-associated viruses included. I would recommend the authors revisit the text to add more substantial details when discussing the literature both in the intro and discussion (info is often very high level/vague vs. citing specific details of the literature). In addition, the report would greatly benefit from text highlighting the limitations of the system (and caveats of data interpretation). I don’t think this would detract from the advancement presented, but would greatly improve the utility of the report, and aid readers in consideration for how these data could be applied and for future studies with these mice.

*Author’s response: We thank the reviewer for acknowledging the importance of the study and useful suggestions. In correspondence with the reviewers detailed suggestions below, we added more substantial details on existing literature in both introduction and discussion, and limitations of our model in the discussion.*

**Reviewer #3**: This manuscript addresses a long-standing issue in the hantavirus field by generating a novel human lung xenograft mouse model that supports infection and replication of HCPS and HFRS-causing viruses. Subcutaneous implantation of human fetal lung tissues in subcutaneous pockets of NSG mice led to successful engraftment and vascularization of xenografts that represented key cell types of adult human lungs, including endothelial cells, epithelial cells, and macrophages, which are targets of hantavirus infection. These xenografts supported the replication of diverse hantaviruses upon intragraft inoculation. Hantavirus infections caused viremia; viruses were disseminated to non-inoculated human lung xenografts and induced localized chemokine and cytokine responses, including IP-10 and RANTES expression. This model can be used for the preclinical evaluation of anti-hantavirus therapeutics as demonstrated by the blockade of Andes virus infection and dissemination by a neutralizing antibody. Although primarily descriptive and hantavirus disease is not recapitulated in this model, this is a well-planned and executed study describing a significant advancement in the field. The manuscript is well-written, and the conclusions are well-supported by the data.

*Author’s response: We thank the reviewer for acknowledging the importance of the study.*

**Part II – Major Issues: Key Experiments Required for Acceptance**
**Reviewer #1**: 
1. The authors provide a good discussion regarding limitations of lung organoids (lines 330-346). However, I lack a discussion about possible limitations of the xenograft mice model compared to organoids, and of possible similarities/differences between these two different systems. This is important as this novel model seems appropriate for studies of certain research questions, such as early replication in a human lung model and test of neutralizing antibodies. However, the xenografts do not function as lungs as there is no air-organ interface, hence the early steps of infection (aerosol transmission and initial infection of target cells in the lung) will not be the same as in human patients. This should be briefly discussed as a limitation of the study. Moreover, as the model is based on immunocompromised mice it needs to be discussed how this model relates to other animal models. For example, most likely lab mice can be asymptomatically infected with hantaviruses, which seems to be verified in this manuscript (Supplementary Fig 5/Lines 180-182) – What is the benefit of this xenograft model based on immunosuppressed mice compared to earlier mice models based on immunocompetent lab mice-strains?

*Author’s response: We thank the reviewer for the useful suggestions. To add more in-depth discussion on the limitations of our current model, we added in the discussion in line 381 the sentence: “However, in our current model the xenografts do not represent fully functional lungs due to the lack of air-organ interface, which limits the translational potential of the model for the study of initial steps of infection including aerosol transmission.” We furthermore added in the discussion in line 389 the sentences: “**Also, as observed during the current study, this mouse model allows for viral spread throughout the murine circulation and different organs. This renders the current model more reproducible and suitable to assess the efficacy of direct countermeasures, such as neutralizing antibodies, that inhibit systemic viral spread, as compared to mouse models based on immunocompetent mice that do not allow for detection of viral antigen throughout the mouse (59). However, the even more important advantage is that the current set-up of the model allows for the study of early events during infection in actual human lung tissue.”*


2. Fig 1D/Line 123 and Fig 5/Line223. Are the resident immune cells/macrophages of human or mice original?

*Author’s response: We did not specifically address this in the current study, as a previous study on a comparable human lung xenograft mouse model has demonstrated by IHC that resident immune cells present in the human lung xenografts are mostly of human origin [Wahl et al. Nat Biotech 2019; reference 37]. In supplementary figure 2, the authors demonstrate the utilization of mouse-specific antibodies and show none to very limited presence of murine hematopoietic cells in the human lung xenografts. In contrast, in supplementary figure 3, the authors demonstrate presence of human CD45^+^ immune cells and human-specific CD68^+^ macrophages (using the same anti-CD68 antibody clone as in the present study) in human lung xenografts. Altogether, this points out that the resident immune cells including macrophages in the human lung xenografts are of human origin.*

3. The finding of hantavirus-specific cytokine responses in the human lung (Lines 231-245) is intriguing, it would be interesting to see levels of human and mouse cytokines in mouse serum/plasma. This could provide more information regarding if the hantavirus-mediated effects on cytokines is limited to the xenografts or if there is also a systemic effect via infected/stimulated mouse cell-produced cytokines. An additional control could be infection of human endothelial cells in vitro with the different hantaviruses for comparison of the results obtained in the xenografts.

*Author’s response: We would like to thank the reviewer for this interesting comment. However, we would like to emphasize that the focus of our model is to study the effects of infection in the human lung xenograft. Although this experiment could provide intriguing results, studying the responses of infection in severely immunocompromised mice, especially regarding the complete knockout of the gamma chain of the interleukin 2 receptor, which results in defective surface receptors (and therefore signalling pathways) for IL-2, IL-4, IL-7, IL-9, IL-15 and IL-21, is beyond the scope of the current study. Also, as one of the main observations of the current study is that more than solely endothelial cells were infected in the human lung xenografts, we do not necessarily agree that repeating our experiment with endothelial monocultures (which lack interactions with other relevant cell types present in our human lung xenografts), would be an adequate control group. For detailed observations of produced cytokines upon orthohantavirus infection, we kindly refer to a recent paper by our group, which analyzed endothelial cell responses by RNA-sequencing [Noack et al. 2024 Lab on a Chip].*
 **Reviewer #2**: No major issues.

**Reviewer #3**: None.

**Part III – Minor Issues: Editorial and Data Presentation Modifications**
**Reviewer #1**: 
1. It should be more clearly stated in the beginning of the manuscript that the model is based on immunosuppressed mice.
*Author’s response: We added this to the sentence in line 127: “Previously, we developed a human lung xenograft mouse model based on severely immunocompromised NOD.Cg-Prkdc^scid^, Il2rg^tm1Wjl^/SzJ (NSG) mice to study Nipah virus infection and early host responses in highly relevant tissue (35).”*

2. In figure legends, include data on how many mice was included in each experiment (as several xenografts can come from the same mouse).
*Author’s response: We have double-checked all figure captions and added this information where needed. We also want to refer to supplementary table 2, where this information is given as an overview for all experimental groups.*

**Reviewer #2**: 
Line 94: provide more details on models that result in disease and those that demonstrate replication in absence of clinical signs/pathology either here or in discussion, as this is relevant to the data presented in the xenograft model. Also, Andes virus was reported to cause disease in immunocompetent hamsters (PMID: 11601912) – this is one of the references listed. If the authors are trying to emphasize models that can be used for both HCPS- “and” HFRS, this can be written more clearly with some more context by providing details of the reports to date.
*Author’s response: We added a more in-depth description of existing literature, starting at line 115 of the introduction: “**Inoculation of HFRS-associated viruses in various tested animal models, such as for example ferrets results in an animal infection model in absence of clinical signs or even detectable viremia (18). While NHP models are restricted by ethical and economic concerns, the immunocompetent Syrian hamster model only displays typical symptoms observed in humans following ANDV inoculation (27) and not following inoculation of other highly pathogenic orthohantaviruses, such as SNV (26) or HTNV (34). The hamster model is also restricted by limited availability of species-specific immunological reagents, but more importantly, must be interpreted carefully due to limited translational potential for the study of relevant human tissues.”*

Line 115: Specify location of subcutaneous implants (dorsum?). For this comment and some that follow, the paper would benefit from adding or moving some of the details from the material and methods to the main text.
*Author’s response: We specified the location of implants accordingly in line 145: “**Human fetal lung tissue was implanted* *into dorsal subcutaneous pockets of NSG mice with a subsequent maturation period of minimal 12 weeks (Fig. 1a), during which easily accessible human lung xenografts developed with a success rate of more than 80%* *(80% of implanted tissues evolving into mature xenografts).”*

Line 144: For clarity, please add more details in the text regarding the study design, including groups sizes, serial timepoints and end of study timepoint.
*Author’s response: We added in line 174 the section: “On 1, 3, 10 and 21 days post inoculation (dpi), animals (N=6) from each experimental group were sacrificed. Two additional control groups were included in which animals were sacrificed at the end of the study at 21 dpi.”*

Line 196: “to variable extents” please provide some more details in the text. Within tissue types, between tissues, between individuals in the experimental group…
*Author’s response: In line 227 we changed the sentence to: “Cells with a positive IHC signal for orthohantavirus antigen were detected for all viruses at all time points, however to variable extents for each experimental group at different time points (Fig. 4a, b, c; Supplementary Fig. 2c, d).”*

Line 247 – 280: based on viral kinetics presented earlier in the paper, the highest levels were typically observed at d21 (the latest timepoint) – is d10 sufficient or would these models require evaluation at later timepoints also? Are there concerns about a “persistent” infection phenotype being observed in the lung xenografts vs. modeling acute infection in “physiologically relevant” tissues?
*Author’s response: We thank the reviewer for this interesting question. Indeed, viral kinetics shown in Figure 3 indicate that on average, highest viral titers are observed 21 days post infection. However, a plateau of the curve, starting from 10 dpi is indicated for majority of tested viruses and also ANDV which was specifically applied in the antiviral treatment. Therefore, we do believe that the choice of 10 days is most relevant for testing of antivirals. Later time points would certainly be of interest for future studies, but were omitted in this proof-of-concept study. The rational of the choice of day 3 and 10 for the study was explained in line 480: “On 3 and 10 dpi, representing time points of initial replication and the beginning of plateaued viral replication, animals from each experimental group (Supplementary Table 3) were euthanized.”*

Line 262: “no viral RNA was found in the sera of KL-AN-5E8 treated animals” – please specify at what timepoints this was assessed
*Author’s response: We apologize for the omission and added ‘at both 3 and 10 dpi’ to the sentence.*

Line 275: Lung xenografts were evaluated in treated mice, were murine tissues also evaluated?
*Author’s response: We did analyse the loads of virus RNA in murine tissues as well, but did not include that figure and information into the manuscript, since it did not change the message about applicability of the xenograft model for the testing of orthohantavirus antivirals. For your reference, we included the results of murine tissues into this rebuttal letter.*

**Detection of ANDV viral loads and antigen in murine tissues in a preclinical set up.**
**a** Heatmap displaying quantification of ANDV S segment RNA loads in murine organs (lungs, kidney, liver and spleen) as determined by RT-qPCR. Each cell represents an individual animal treated with KL-AN-5E8 or CR9114 (sham) expressed in TCID_50_ equivalents per gram tissue. Grey cells represent that a sample was below lower limit of detection. **b** Quantification of virus antigen detection in murine lungs was performed by counting the number of positive cells for antigen staining per ten high power fields (HPFs). Each circle indicates the murine lungs of one evaluated animal. Bars represent the mean and error bars represent the standard error of the mean.  Number of virus antigen-positive cells in murine lungs in both treatment groups were compared per time point with a two-tailed Mann Whitney U test. *p<0.05.

Line 365, 367: should be “Tecniplast” without the “h”?
*Author’s response: We corrected the typing error in the text.*

Line 369: “Sterilized” food and water?
*Author’s response: We added further details in line 419: “Irradiated food and acidified water were available ad libitum.”*

Line 373: clarify if mice were euthanized by exsanguination and/or isoflurance overdose (followed by cervical dislocation?)
*Author’s response: We clarified accordingly in line 423: “Mice were euthanized by cardiac puncture and exsanguination.”*

Line 375: Were inocula backtitered? Please provide target and backtiter doses
*Author’s response: We would like to refer to supplementary table 1, where the backtiters are displayed. The method of TCID50 assay is identical to the description in the methods section ‘virus titer quantification with endpoint dilution assay’, which mentions that the target for immunofluorescence detection is orthohantavirus N protein 5 days post-inoculation of Vero E6 cells.*

Discussion: Please add a section of limitations of the model, caveats to data interpretation, thoughts on replication kinetics (what timepoints
*Author’s response: We thank the reviewer for the useful suggestions. To add more in-depth discussion on the limitations of our current model, we added in the discussion in line 381 the sentence: “However, in our current model the xenografts do not represent fully functional lungs due to the lack of air-organ interface, which limits the translational potential of the model for the study of initial steps of infection including aerosol transmission.” Also, we added in the discussion in line 389 the sentences: “Also, as observed during the current study, this mouse model allows for viral spread throughout the murine circulation and different organs. This renders the current model more reproducible and suitable to assess the efficacy of direct countermeasures, such as neutralizing antibodies, that inhibit systemic viral spread, as compared to mouse models based on immunocompetent mice that do not allow for detection of viral antigen throughout the mouse (59).”*

Fig 3: Please clarify group sizes in the figure legend as only mean is depicted (group sizes would be helpful in other legends also, but more critical for this one).
*Author’s response: We have double-checked all figure captions and added this information where needed. We also want to refer to supplementary table 2, where this information is given as an overview for all experimental groups.*

Other:
-Sundström et al. reported on virus kinetics and associated cytokine response in a 3D Human Lung Tissue Model (PMID: 26907493; PMCID: PMC4764364). This seems like a potentially overlooked relevant reference to include in this paper?
*Author’s response: We added a sentence in line 353 on this particular study: “Also, one of the small number of in vitro studies that investigated ANDV infection in human lung cells using a co-culture model of human bronchial epithelial cells and lung fibroblast cell line, demonstrated increases in pro-inflammatory markers such as IP-10, but also IL-6 and IL-8 (53).”*

-There are many references on cytokine analyses in cells, animal models and humans – it would strengthen the paper to discuss if how these results are similar or differ to previous studies and the resultant clinical relevance.
*Author’s response: We added a section in line 339: “**Infection of these cells results in increased expression of key inflammatory mediators IP-10 and RANTES by multiple orthohantaviruses, as observed previously in vitro (47) and* *are also in line with the observed upregulation of genes encoding IP-10 (CXCL10) and RANTES (CCL5) in ANDV-inoculated hamster models that recapitulate human HCPS symptoms (34). IP-10 can be expressed by different cell types, like endothelial cells, and can act as chemoattractant for macrophages, T cells, natural killer cells and dendritic cells, which all have been described to play an important role in orthohantavirus pathogenesis (2, 48, 49). Similar as in our model, IP-10 is increased in human HCPS cases (50, 51) and in HFRS patients (50, 52), but not in HTNV-inoculated hamsters, which did not demonstrate significant increased expression of CXCL10 (34), as in our described model.”*

 **Reviewer #3**: 
Include a sentence about the success rate of xenografting.
Author’s response: *We included a sentence about the success rate in line 145: “Human fetal lung tissue was implanted into dorsal subcutaneous pockets of NSG mice with a subsequent maturation period of minimal 12 weeks (Fig. 1a), during which easily accessible human lung xenografts developed with a success rate of more than 80%* *(80% of implanted tissues evolving into mature xenografts).”*

Fig 1b - Add scale bar. Author’s response: *We added a scale bar to figure 1b.*

Fig S2 legends - “azoom-in” should be “a zoom-in” Author’s response: *We corrected the typing error in figure caption of S2.*

Furthermore, we have used track changes to remove some minor typographical mistakes that we have spotted ourselves. These have no effect on any of the scientific results.

Sincerely yours, also on behalf of the co-authors,

Melanie Rissmann
